# Analysis of Choriocapillaris Reperfusion Topography following Faricimab Treatment for Neovascular Age-Related Macular Degeneration in Non-Treatment-Naïve Patients

**DOI:** 10.3390/diagnostics14090901

**Published:** 2024-04-25

**Authors:** Max Brinkmann, Pasquale Viggiano, Giacomo Boscia, Mathis Danckwardt, Evelyn Susantija, Tom Müller, Niccolò Castellino, Jakob Schweighofer, Francesco Boscia, Mario Damiano Toro, Yosuf El-Shabrawi

**Affiliations:** 1 Department of Ophthalmology, Klinikum Klagenfurt, 9020 Klagenfurt, Austria; tom.mueller@kabeg.at (T.M.);; 2 Department of Ophthalmology, Universitätsklinikum Schleswig-Holstein, 23564 Lübeck, Germany; mathis-danckwardt@web.de (M.D.); esusantija@gmail.com (E.S.); 3 Department of Translational Biomedicine Neuroscience, University of Bari “Aldo Moro”, 70121 Bari, Italy; pasquale.viggiano90@gmail.com (P.V.); bosciagiacomo@gmail.com (G.B.); francescoboscia@hotmail.com (F.B.); 4 Department of Ophthalmology, University of Catania, 95123 Catania, Italy; ncastellino7@gmail.com; 5 Department of Ophthalmology and Optometry, Medical University of Vienna, 1090 Vienna, Austria; jakob.schweighofer@meduniwien.ac.at; 6 Eye Clinic, Public Health Department, University of Naples Federico II, 80133 Naples, Italy; 7 Chair and Department of General and Pediatric Ophthalmology, Medical University of Lublin, 20079 Lublin, Poland

**Keywords:** faricimab, choroid, OCTA, anti-VEGF, angiopoietin 2, AMD

## Abstract

To assess changes in choriocapillaris (CC) vascular density surrounding macular neovascularization (MNV) in age-related macular degeneration (AMD) when transitioning from various anti-VEGF treatments to faricimab, using optical coherence tomography angiography (OCTA). 25 eyes of 22 individuals who underwent intravitreal faricimab injections for neovascular AMD with type 1 MNV were included. OCTA images were obtained prior to (T0), after one (T1), and after three faricimab injections (T2); Noteworthy changes occurred in the first ring at T2 in comparison to T0. The percentage of CC flow deficit (FD%), FD average area (FDa), and FD number (FDn) in 5 rings (R1-R5) surrounding the dark halo around the MNV were calculated. A reduction in FD% at T2 compared to T0 (50.5 ± 10.2% at T0, 46.4 ± 10.6% at T2; *p* = 0.020) was seen, indicating CC reperfusion. Additionally, we observed a reduction in the average FDa (140.2 ± 172.1% at T0, 93.7 ± 101.8% at T2; *p* = 0.029). Our study highlights an FD% after three consecutive faricimab injections. The most pronounced effect was observed in the first ring, directly adjacent to the dark halo, suggesting a partial CC reperfusion surrounding the MNV, potentially indicating disease regression.

## 1. Introduction

Age-related macular degeneration (AMD) stands as a prominent cause of blindness, impacting over 6 million individuals worldwide [1,2,3]. There exists compelling evidence suggesting a pivotal role of impaired choroidal flow signal in the pathophysiology of AMD. Recent histopathologic examinations have unveiled instances of choroidal dysregulation, even in the early and intermediate stages of AMD, promoting angiogenesis and continuous vascular remodeling [4,5,6,7]. Notably, heightened impairment in choroidal, particularly choriocapillaris (CC) flow, is significantly associated with AMD-afflicted eyes compared to non-AMD eyes [8]. This impairment is particularly pronounced in neovascular AMD (nAMD), where irregularities in the spatial distribution of the CC align with the development of abnormal blood vessels in the sub-retinal pigment epithelium (RPE) cell spaces, leading to the occurrence of macular neovascularization (MNV) [6]. The spatial distribution of CC impairment surrounding MNV can be quantified by delineating the MNV and measuring the flow deficit (FD) at specific distances from the MNV outline [6,9].

Vascular endothelial growth factor (VEGF) secreted by the RPE plays another key role in the formation of MNV [10,11]. Up until recently, VEGF was the key protein to target in several diseases involving MNV formation [11,12]. Knowledge about the impact of anti-VEGF drugs on the choroid is limited and controversial. Some studies reported that long-term administration of anti-VEGF agents may promote decreased vascular density, especially in the CC [13,14]. Using optical coherence tomography (OCT) and OCT angiography (OCTA) previous studies have shown that intravitreally applied anti-VEGF agents have an impact on the choroid, including CC flow signal [15,16,17]. Viggiano et al. analyzed this effect, and observed that FD decreased and CC surrounding the MNV was reperfused after loading anti-VEGF therapy. 

In 2022, the Food and Drug Administration (FDA) marked a noteworthy advancement in the treatment of nAMD and diabetic macular edema [18]. Distinguished as the first intraocular drug sanctioned by the FDA to target both VEGF and angiopoietin2 (Ang2), faricimab holds promise in addressing these ocular conditions. Ang2, a crucial component of the Ang/Tie pathway, plays a multifaceted role in vascular homeostasis, influencing vascular permeability and participating in neoangiogenic and proinflammatory processes [19,20]. Despite its recognized impact on these processes, the specific influence of Ang2 on choroidal flow signal remains unexplored in vivo. This aspect is particularly intriguing, given Ang2’s role in regulating vascular homeostasis. As a result, this research aims to evaluate the choroidal flow signal before and after the intravitreal injection of faricimab utilizing OCTA.

## 2. Materials and Methods

In this retrospective study, we assessed 25 eyes from 22 individuals who received intravitreal faricimab injections for neovascular AMD and type 1 MNV at the Department of Ophthalmology, Klinikum Klagenfurt, Austria. The period of investigation spanned from November 2022 to October 2023. Prior to the switch to faricimab, all patients had previously received treatment with other anti-VEGF agents, specifically: aflibercept, ranibizumab, and brolucizumab. The study adhered to the principles outlined in the Declaration of Helsinki and received approval from the local review board (Ethikkommission Kärnten, S2023-13). All subjects provided informed consent prior to treatment. As data were deidentified, consent for publication was not required. 

Each patient underwent a comprehensive ophthalmologic assessment, encompassing the measurement of best-corrected visual acuity (BCVA), intraocular pressure (IOP), and dilated ophthalmoscopy. Only individuals with IOP levels falling within the normal range (10–21 mmHg) were considered for inclusion. Exclusion criteria comprised: (i) significant cataract; (ii) myopia exceeding 3.00 diopters; (iii) occurrence of myocardial infarction or cerebrovascular disease within the preceding 6 months; (iv) presence of infection or inflammation in both eyes; (v) existence of other concurrent retinal and/or macular conditions (e.g., diabetic retinopathy and retinal venous occlusion); (vi) any form of optic neuropathy, including glaucoma and (vii) presence of type 2 or type 3 MNV. 

**Figure 1:**
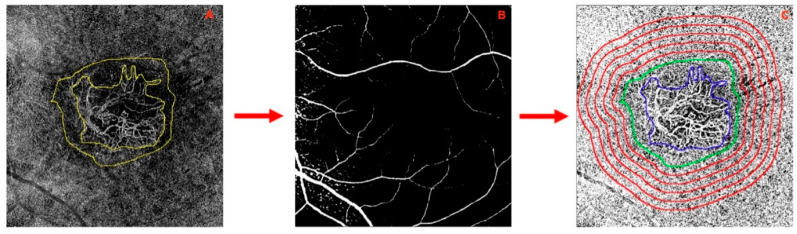
(**A**–**C**) Analysis of choriocapillaris (CC) using optical coherence tomogra-phy angiography en-face imaging. (**A,B**) Automated segmentation was employed to extract 20 µm slabs of the CC, with exclusion of CC areas beneath major superficial retinal vessels to mitigate potential shadows or projection artifacts. (**C**) The 6 × 6 mm en face CC image showcases subfoveal treatment-naïve macular neovascularization (MNV, highlighted in blue). The perilesional dark halo, denoted by a low-flow area surrounding the MNV, is encircled in green.

ZEISS PLEX Elite 9000 Swept-Source OCT Angiography (Carl Zeiss AC, Jena, Germany) was used to capture patient imaging data. OCTA imaging occurred at three specific time points: the “T0 visit”, representing 0 to 3 days before the initial injection of Faricimab; the “T1 visit”, corresponding to the examination conducted one month after the T0 visit; and the “T2 visit”, corresponding to four weeks following the third consecutive application of faricimab. En face, OCTA 6 × 6 mm volume scans were obtained. Each imaging session comprised OCTA volumetric scans of the posterior pole. The device’s follow-up mode ensured consistent measurements at identical locations across all time points. OCTA scans with a strength index below eight out of ten or exhibiting significant motion artifacts or shadowing effects were excluded from the analysis [21]. As part of our clinic’s protocol, all medical retina patients underwent routine assessments during a specified time frame (between 08:00 and 12:00 a.m.), mitigating potential bias arising from physiological diurnal changes in ocular flow signal [22,23]. Manufacturer default settings were employed to automatically segment OCTA images in all B-scans. Automated segmentation facilitated the extraction of 20 µm slabs of the CC. CC areas beneath major superficial retinal vessels were excluded from the analysis to prevent potential shadows or projection artifacts [24] (Figure 1). Additionally, for inclusion in the study, all edges of the MNV were required to be localized at least 1 mm away from the scan edge.

A subsequent analysis was carried out following a previously established protocol designed for the quantification of flow metrics [17,25]. En-face OCTA images of the choriocapillaris were imported into Fiji ImageJ (software version 2.0.0; National Institute of Health, Bethesda, MD, USA). The borders of the MNV lesion and the associated dark halo (DH) were manually outlined by two masked expert graders (authors PV and GB) in each en face CC OCTA scan (Figure 1A). 

**Figure 2:**
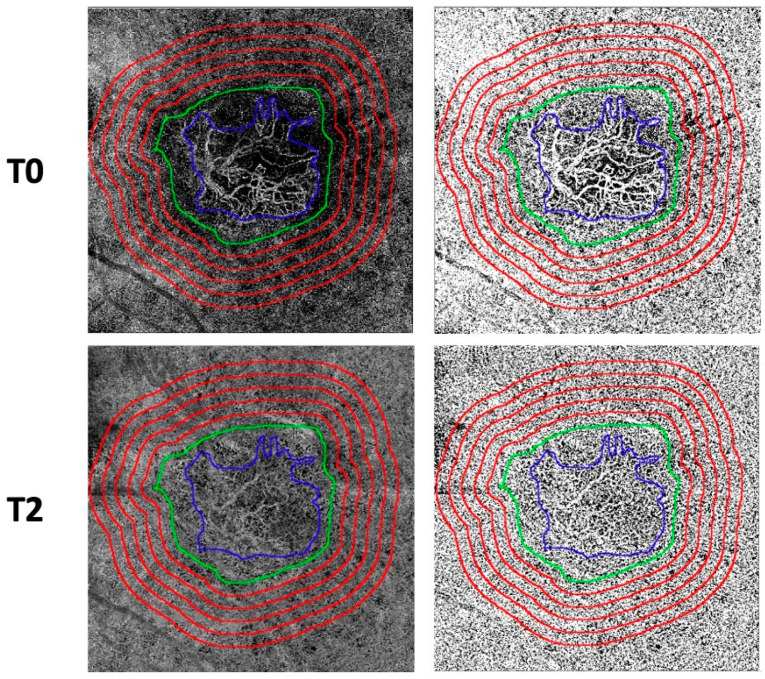
En-face choriocapillaris (CC) optical coherence tomography angi-ography images are employed for the manual delineation of borders for both the MNV lesion and its associated dark halo. Subsequently, utilizing the “Distance Map” function in ImageJ, five progressive concentric rings, each 200 μm wide, are generated from the periphery of the dark halo’s edge. This function in ImageJ automatically establishes a border that follows the contour of the perilesional halo. Each ring (R1, R2, R3, R4, and R5) was incorporated into the Region of Interest (ROI) manager for CC flow analysis. This custom configuration, unique to each patient, was then implemented on the CC en-face images at both T1 and T2, maintaining consistent size and position for com-parative analysis. Subsequently, the obtained CC images underwent binarization for the quantitative assessment of the FD within each ring. The Phansalkar method was employed for this purpose.

A low-flow signal area surrounding each MNV, known as the dark halo (DH), was identified [17]. Each CC image underwent compensation to eliminate retinal vessel projection artifacts and adjustment for shadowing artifacts [25] (Figure 1B). Continuing, we generated five progressive 200-μm-wide concentric rings from the DH edge using the “Distance Map” function in ImageJ, which automatically creates a border that follows the contour of the perilesional halo. (Figure 1C). Each ring (R1, R2, R3, R4, and R5) was added to the region of interest (ROI) manager for CC flow analysis. The custom configuration, unique for each patient, consisting of these rings, was applied to the CC en face at T0, T1, and T2 at the same size and position (Figure 2) [26].

Subsequently, the resulting CC images were binarized using the Phansalkar method for the quantitative measurement of the FD in each ring using a radius of 15 pixels (Figure 2) [27]. The “analyze particles” command was employed to calculate the CC FD, and the “Analyze Particles” tool provided by ImageJ was used to quantify CC flow in each ring area (R1, R2, R3, R4, and R5). Specifically, the following metrics were quantified: (i) the FD percentage (FD%), representing the percentage of flow deficits within the analyzed area; (ii) the FD average area (FDa), representing the average size of the flow deficits within the analyzed region; and (iii) the FD number (FDn), quantifying the number of flow deficits in the ROI.

Statistical calculations were conducted using the Statistical Package for Social Sciences (SPSS IBM Statistic 25, Chicago, IL, USA). The data distribution was assessed using the Shapiro–Wilk test. Paired *t*-tests were used to analyze quantitative data across the study visits. A significance level of *p* < 0.05 was chosen to determine statistical significance.

## 3. Results

A total of 25 eyes of 22 Caucasian patients receiving a series of at least two faricimab injections (mean 3.3 ± 1.6) for the treatment of nAMD were included in the study. Due to poor treatment response, all patients were switched from other anti-VEGF agents (ranibizumab, aflibercept and brolucizumab). On average, patients had received 15.9 ± 15.3 intravitreal injections with anti-VEGF drugs before being switched to faricimab (range 2–53, ±SD; Table 1). During the former treatment regimen, the latest time interval between injections was 5.4 ± 1.1 weeks (range 4–8, ±SD; Table 1). A total of 17 patients were female, 5 patients were male. The cohort consisted of 12 right and 13 left eyes. The mean age was 78.4 ± 6.6 years (range= 64–89 years; ±SD). Mean BCVA was 0.50 ± 0.3 (range 0.05–1.0 LogMAR, ±SD) at T0, 0.47 ± 0.33 (range 0.1–1.0 LogMAR, ±SD) at T1, and 0.59 ± 0.34 (range 0.1–1.0 LogMAR, ±SD) at T2. In the subjects included in the analysis, the MNV lesion displayed an average mean area of 0.23 ± 0.33 mm^2^ at T0, 0.20 ± 0.45 mm^2^ at T1, and an average mean area of 0.15 ± 0.20 mm^2^ at T2. Interobserver agreement (average) was found to be excellent in the MNV assessment (0.90 (confidence interval, 0.86–0.93). The characteristics of the subjects included in the analysis are summarized in Table 1.

The topographical CC subanalysis revealed statistically significant changes in the first ring at T2 compared to T0. In specific terms, R1 exhibited a progressive reduction in FD% at T2 compared to T0 values (50.5 ± 10.2% at T0 and 46.4 ± 10.6% at T2; *p* = 0.020), signifying a gradual CC reperfusion within this ring (Table 2).

Similarly, the average size of FD was significantly lower at T2. Notably, we observed a gradual contraction in the average FD area within R1 (140.2 ± 172.1 at T0 and 93.7 ± 101.8% at T2; *p* = 0.029) (Table 3).

Conversely, the topographical analysis of the CC using OCTA did not reveal any statistically significant changes at T1 when compared to T0 within all the analyzed rings. Our findings indicated no significant alterations in terms of FD%, FDa, and FDn (Table 2, Table 3 and Table 4).

## 4. Discussion

The primary objective of this study was to assess the CC flow changes using OCTA in eyes treated for neovascular AMD when the treatment was switched to faricimab. Prior research has underscored the significance of the choroid, particularly the CC, in the context of AMD [4,7,28,29]. It is hypothesized that dysfunction of the CC plays a pivotal role in the initiation and progression of AMD [7,29,30]. Considering that faricimab is the first approved drug targeting VEGF and Ang2, investigating its effects on CC flow signal holds particular interest.

Our findings indicate a significant decrease in perilesional CC flow density following faricimab treatment. At T2, there was a noteworthy increase in CC reperfusion, particularly in the first ring immediately adjacent to the DH. Specifically, our results demonstrate a significant reduction in FD% and a contraction of FDa.

Numerous studies have provided evidence supporting CC impairment in the context of AMD. Histological findings indicate that CC degeneration has an impact on the viability of the RPE [29]. Biesemeier et al., using light and electron microscopy, observed that CC breakdown is a normal part of aging but becomes significantly more pronounced when AMD develops [4]. They further noted that CC alterations precede RPE loss, leading to the conclusion that AMD can be characterized as a vascular disease [4].

Spaide, in an examination involving 104 eyes of 80 AMD patients using OCTA, described significant alterations in the flow pattern [31]. In a separate cohort of 42 eyes with intermediate AMD, Borrelli et al. later discovered that eyes with intermediate AMD in patients with nAMD in the fellow eye exhibited an increased average size of CC signal voids compared to eyes without nAMD in the fellow eye [7].

Ang2 is a component of the Ang/Tie pathway, playing a role in the regulation of vascular homeostasis, modulation of vascular permeability, and involvement in neoangiogenic and proinflammatory processes [19]. The angiogenic or anti-angiogenic activity of Ang2 depends on the context, with one of the determining factors being the expression of other angiogenic growth factors, notably VEGF [32]. In vitro studies have indicated that Ang2 induces permeability and angiogenesis on the pupillary membrane when VEGF is present. However, in the absence of VEGF, Ang2 leads to vessel regression and endothelial cell death [33]. While caution is warranted in extrapolating these findings to clinical scenarios, it is conceivable that blocking Ang2, especially in conjunction with VEGF depletion, could impact the permeability of choroidal vessels, indirectly influencing CC perfusion. Razavi et al. have reported a similar observation, speculating that in nAMD, anti-VEGF treatment may not only positively affect retinal exudation but also underlying choroidal exudation by reducing choroidal vascular hyperpermeability [34].

No notable change in BCVA was observed in our study. This lack of significant change is likely attributed to our cohort’s advanced stage of nAMD. For this analysis, we specifically included patients who had transitioned from other anti-VEGF agents to faricimab. Many of these individuals had received prolonged treatment over several years, leading to advanced morphological changes. This constitutes a noteworthy limitation of our study, as these advanced morphological changes may also impact choroidal health.

As of our current understanding, this study represents the initial exploration into the influence of intravitreally administered faricimab on CC flow. It is essential to acknowledge the limitations inherent in the retrospective design and the relatively small sample size of the study. Additionally, all participants had prior treatment history with other anti-VEGF agents. Measuring CC flow and FD using OCTA is challenging, especially in AMD’s setting. There is an ongoing discussion about potential errors caused by technical and anatomical conditions [35]. We have addressed this issue by applying an up-to-date acquisition and image processing method, which has proven robust with an excellent repeatability in several published studies [25,36]. This method has shown to be effective in compensating for the signal loss caused by retinal vessels and the RPE/Bruch’s membrane complex. It was especially tested to account for the shadowing effect from the elevated RPE caused by Drusen in the setting of AMD [25]. Adaptive local thresholding was performed to obviate small regional variations in image brightness, and the Phansalkar method was used because it was designed to select darker regions in potentially low-contrast images [27]. Moreover, we used a swept-source OCTA device that allowed for deeper light penetration into the choroid, thereby obtaining higher-quality images with outstanding repeatability [25,37].

## 5. Conclusions

In conclusion, our study reports a decrease in CC FD after the administration of a series of three faricimab injections. This effect was greatest in the first ring immediately adjacent to the dark halo. This finding indicates at least partial reperfusion of the CC surrounding the MNV, which may be an indicator of disease regression. In the future, larger prospective studies are needed to support our preliminary results.

## Figures and Tables

**Table 1:** The clinical characteristics of subjects included in the analysis.

	MNV Type 1 Eyes (*n* = 25)		
Age (years)	78.4 ± 6.6		
Gender (female, %)	17 (68%)		
Right eye, *n*	12		
injections per eye	15.9 ± 15.3		
ranibizumab	13.8 ± 12.8		
aflibercept	5.1 ± 3.4		
brolucizumab	10 ± 0		
interval between injections (weeks)	5.4 ± 1.1		
	T0	T1	T2
MNV area (mm^2^)	0.52 ± 0.12	0.48 ± 0.13 *p* = 0.049 ^a^	0.49 ± 0.13 *p* = 0.367 ^a^ *p* = 0.740 ^b^
BCVA logMAR	0.50 ± 0.3	0.47 ± 0.33 *p* = 0.58 ^a^	0.59 ± 0.34 *p* = 0.46 *p* = 0.19

Data are presented as Mean ± SD. MNV macular neovascularization; BCVA best-corrected visual acuity; ^a,b^ Paired test, ^a^ comparison with T0; ^b^ comparison with T1.

**Table 2:** OCT angiography FD % data and comparisons.

	CC FD (%) T0	CC FD (%) T1	CC FD (%) T2
Ring 1 (R1)	50.5 ± 10.2	47.8 ± 11.4	46.4 ± 10.6
		0.362 ^a^	0.020 ^a^
			0.704 ^b^
Ring 2 (R2)	44.5 ± 9.6	42.2 ± 9.2	41.7 ± 9.3
		0.183 ^a^	0.602 ^a^
			0.862 ^b^
Ring 3 (R3)	39.7 ± 8.6	38.9 ± 7.9	38.3 ± 7.2
		0.552 ^a^	0.739 ^a^
			0.615 ^b^
Ring 4 (R4)	36.9 ± 7.5	37.1 ± 7.7	35.6 ± 5.6
		0.881 ^a^	0.975 ^a^
			0.839 ^b^
Ring 5 (R5)	34.9 ± 6.8	35.5 ± 7.5	33.3 ± 4.8
		0.432 ^a^	0.764 ^a^
			0.732 ^b^

Data are presented as Mean ± SD. FD% flow deficit percentage MNV macular neovascularization; ^a,b^ Paired test, ^a^ comparison with T0; ^b^ comparison with T1.

**Table 3:** OCT angiography FDa; data and comparisons.

	CC FDa T0	CC FDa T1	CC FDa T2
Ring 1 (R1)	140.2 ± 172.1	129.5 ± 159.1	93.7 ± 101.8
		0.733 ^a^	0.029 ^a^
			0.411 ^b^
Ring 2 (R2)	62.6 ± 53.5	76.4 ± 17.6	49.7 ± 41.5
		0.665 ^a^	0.021 ^a^
			0.337 ^b^
Ring 3 (R3)	39.4 ± 26.1	37.5 ± 22.1	35.1 ± 19.7
		0.934 ^a^	0.380 ^a^
			0.445 ^b^
Ring 4 (R4)	30.4 ± 17.5	28.7 ± 14.2	28.9 ± 12.4
		0.541 ^a^	0.919 ^a^
			0.481 ^b^
Ring 5 (R5)	26.2 ± 15.7	24.4 ± 11.6	26.8 ± 20.8
		0.279 ^a^	0.581 ^a^
			0.861 ^b^

Data are presented as Mean ± SD. FDa flow deficit average area; ^a,b^ Paired test, ^a^ comparison with T0; ^b^ comparison with T1.

**Table 4:** OCT Angiography FDn; Data and Comparisons.

	CC FDn T0	CC FDn T1	CC FDn T2
Ring 1 (R1)	895.1 ± 1267.4	956.5 ± 1421.2	1312.7 ± 1363.1
		0.238 ^a^	0.067 ^a^
			0.386 ^b^
Ring 2 (R2)	654.3 ± 474.1	643.1 ± 505.7	833.7 ± 643.3
		0.254 ^a^	0.150 ^a^
			0.341 ^b^
Ring 3 (R3)	861.1 ± 537.2	886.8 ± 577.9	959.1 ± 500.1
		0.304 ^a^	0.504 ^a^
			0.338 ^b^
Ring 4 (R4)	1121.2 ± 621.5	1159.1 ± 666.1	1140.6 ± 506.1
		0.332 ^a^	0.862 ^a^
			0.334 ^b^
Ring 5 (R5)	1309.4 ± 588.8	1337.3 ± 624.1	1263.6 ± 493.1
		0.343 ^a^	0.448 ^a^
			0.324 ^b^

Data are presented as Mean ± SD. FDn flow deficit number MNV, CC choriocapillaris; ^a,b^ Paired test, ^a^ comparison with T0; ^b^ comparison with T1.

## Data Availability

The data presented in this study are available on reasonable request from the corresponding author. The data are not publicly available due to patient privacy.
